# Fabrication and Characterization of the Micro-Heater and Temperature Sensor for PolyMUMPs-Based MEMS Gas Sensor

**DOI:** 10.3390/mi13040525

**Published:** 2022-03-26

**Authors:** Abdullah S. Algamili, Mohd Haris Khir, Abdelaziz Y. Ahmed, Almur A. Rabih, Saeed S. Ba-Hashwan, Sami S. Alabsi, Osamah L. Al-Mahdi, Usman B. Isyaku, Mawahib G. Ahmed, Muhammad Junaid

**Affiliations:** 1 Department of Electrical and Electronic Engineering, Universiti Teknologi PETRONAS, Bandar Seri Iskandar 32610, Malaysia; harisk@utp.edu.my (M.H.K.); saeedsb2013@gmail.com (S.S.B.-H.); sami_18001518@utp.edu.my (S.S.A.); osamaal-mahdi@hotmail.com (O.L.A.-M.); usman_1800411@utp.edu.my (U.B.I.); 2 Department of Engineering, Thamar University, Dhamar 124401, Yemen; 3 Département de Génie éLectrique, École de Technologie Supérieure, 1100 Notre-Dame St W, Montreal, QC H3C 1K3, Canada; azez23101@gmail.com (A.Y.A.); almurutp@gmail.com (A.A.R.); 4 Applied Physics Electrical and Instrumentation, Faculty of Engineering & Technology, University of Gezira, Wad Medani 12217, Sudan; mawahib126@yahoo.com; 5 Department of Electronic Engineering, Balochistan University of Information Technology, Engineering and Management Sciences, Quetta 87300, Pakistan; muhammad_17000796@utp.edu.my

**Keywords:** characterization, fabrication, gas detection, MEMS devices, PolyMUMPs sensor

## Abstract

This work describes the fabrication and characterization of a Micro-Electro-Mechanical System (MEMS) sensor for gas sensing applications. The sensor is based on standard PolyMUMPs (Polysilicon Multi-Users MEMS Process) technology to control the temperature over the sensing layer. Due to its compact size and low power consumption, micro-structures enable a well-designed gas-sensing-layer interaction, resulting in higher sensitivity compared to the ordinary materials. The aim of conducting the characterization is to compare the measured and calculated resistance values of the micro-heater and the temperature sensor. The temperature coefficient of resistance (TCR) of the temperature sensor has been estimated by raising and dropping the temperature throughout a 25–110 °C range. The sensitivity of these sensors is dependent on the TCR value. The temperature sensor resistance was observed to rise alongside the rising environmental temperatures or increasing voltages given to the micro-heater, with a correlation value of 0.99. When compared to the TCR reported in the literature for the gold material 0.0034 °C${}^{-1}$, the average TCR was determined to be 0.00325 °C${}^{-1}$ and 0.0035 °C${}^{-1}$, respectively, indicating inaccuracies of 4.6% and 2.9%, respectively. The variation between observed and reported values is assumed to be caused by the fabrication tolerances of the design dimensions or material characteristics.

## 1. Introduction

Gas sensors with high sensitivity and low-temperature capabilities are in high demand [1]. Gas sensors are essential for monitoring and detecting harmful gases, assuring safety and air quality, bio-medical monitoring, and improving lifestyle quality [2,3]. Sensors based on semiconductor-metal oxides, including (SnO_2_) tin dioxide, (TiO_2_) titanium dioxide, and (ZnO) zinc oxide sensors, have recently become the most preferred alternative for gas detection [4,5]. Nevertheless, the exceptional sensitivity of these sensors could only be achieved at rather high temperatures (usually between 300 and 450 °C), implying an increase in power consumption. Furthermore, there are selectivity limitations [6]. As a result, developing reliable, miniature, and precise sensors to detect and monitor harmful gases in various applications is widely sought nowadays. Implementing gas-sensing materials in micro- or nano-structured form is one of the most effective approaches to enhance their performances [7]. Furthermore, the relatively low price and ease of fabrication play a significant role in commercial production [8].

Several efforts are being conducted to develop MEMS-based sensors for the application of gas detection. Micro-electro-mechanical systems (MEMSs) are tiny devices that enable complicated systems to operate [9,10]. Because of its advantages, such as small size, good performance, mass-fabrication capacity, and being relatively inexpensive, the MEMS has achieved widespread utilization [11,12,13,14]. Today, they may be found in various applications, including industrial, aerospace, bio-medical, and automotive [15,16,17]. Despite the gas and pressure sensors used in the bio-medical and automotive industries being the earliest industrial uses for MEMS devices, the MEMS is now employed in a wide range of other industry areas [18,19]. Recently, MEMS technology has been utilized to fabricate a variety of sensor technologies.

The micro-heater is the key component of the majority of MEMS sensors used in gas sensing applications. They are used in conjunction with gas sensors to deliver the proper temperature to the film that controls the sensitivity of such sensors [20]. They are also utilized to speed up the adsorption process between the sensitive layers and also the gas species being detected [21]. Noble metals such as platinum, aluminum, and gold are thought to be ideal for designing micro-heaters that achieve high precision, a wide range of temperature, and stability. In gas sensors, a micro-heater is employed to control the temperature on the surface of the sensing layer. Many studies have reported the usage of various materials on a heater layer, such as aluminum [22], platinum [23], and polysilicon [24].

This work describes the fabrication and characterization of a micro-heater and the temperature sensor using a MEMS-based PolyMUMPs sensor proposed for gas sensing applications. Due to the sensitivity of these sensors being dramatically increased at higher temperatures, the uniformity of the temperature gradient throughout the sensing plate is a critical component in gas detection. Micro-heaters can provide excellent heat uniformity, a quick response, and low power consumption, making them ideal for use as a MEMS-based PolyMUMPs gas sensor.

## 2. Design of the PolyMUMPs Sensor

**Figure 1:**
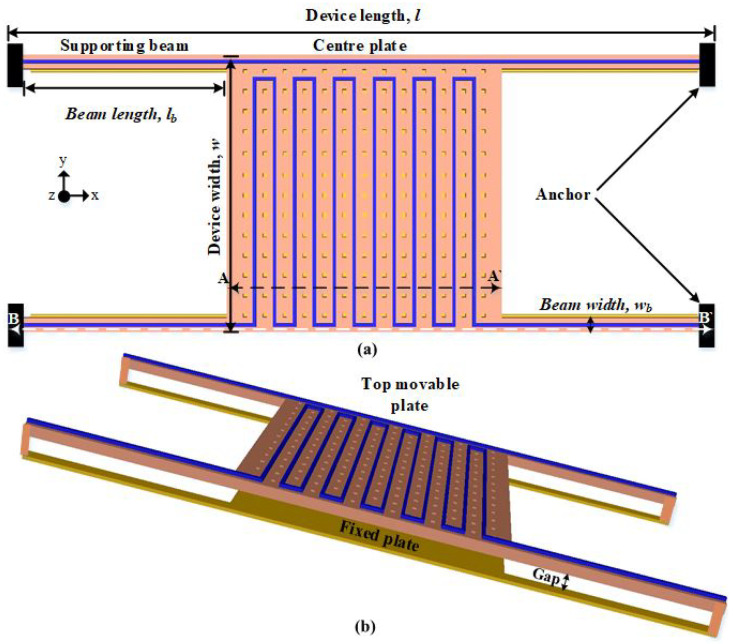
The PolyMUMPs sensor’s schematic showing (**a**) the top movable plate and (**b**) 3D schematic representation [18].

Figure 1 shows a 3D model of the suggested sensor schematic designed using the PolyMUMPs fabrication process. The PolyMUMPs sensor consists of two square plates with (400 μm × 400 μm) dimensions, namely the upper plate (the vibrating part) and the bottom plate (stator). The upper plate, which is supported by four beams, is a movable part fabricated of Poly2 and a gold metal layer of a total thickness 2 μm. The other plate is a Poly0 bottom plate that is attached to the silicon substrate and made of Poly0. To create a parallel plate capacitor for sensing, the first polysilicon layer is removed, producing a 4.75 μm gap between the two plates. A gold metal layer is used to create the actuation micro-heater, with a temperature sensor on the top moving plate. It has etched holes to allow the device to be released and to reduce squeeze-film dampening between the fixed and moving plates. Actuation is achieved electro-thermally through the micro-heater.

**Figure 2:**
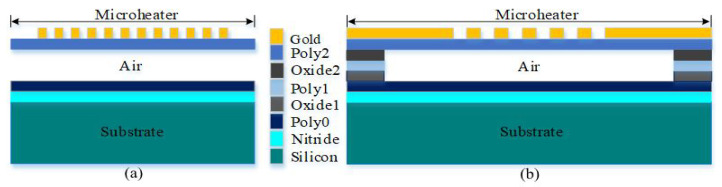
The PolyMUMPs process’s schematic of (**a**) the cross-section along AA’ view of the center plate and (**b**) the cross-section along BB’ view.

Comprehensive analytical modeling and finite element analysis (FEA) utilizing the 2008 CoventorWare simulation program were used to determine the optimum dimensions of the various components of the PolyMUMPs sensor. The dimensions of the PolyMUMPs sensor are listed in Table 1. Figure 2 shows the cross-sectional view along AA’ and BB’.

## 3. Designing of the Micro-Heater and the Temperature Sensor

**Figure 3:**
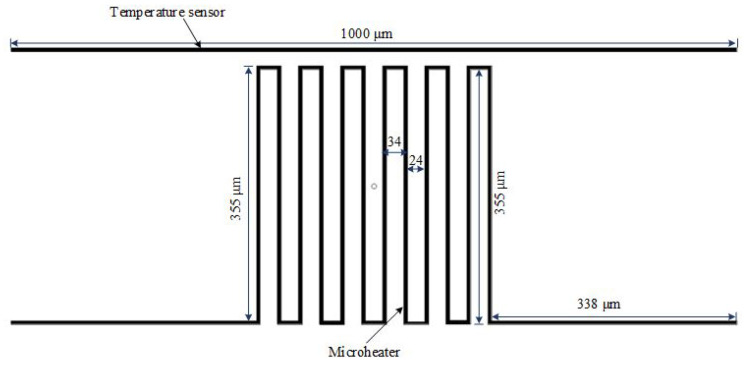
Structure and dimension of the micro-heater and the temperature sensor.

The micro-heater is considered the main part of lots of the MEMS devices used in gas sensing applications. Actuation is achieved electro-thermally through a micro-heater that produces the required heat due to applying a certain current to a resistor. They are used in conjunction with metal oxide gas sensors to supply the proper temperature to the film, which determines the sensor’s sensitivity and selectivity [20]. They are also employed to speed up the process of adsorption between the sensitive layers and the gas species being detected [21]. Micro-heaters made of noble metals such as platinum and gold are said to have the best temperature range, precision, and stability. Gold metal was used to design the micro-heater and the temperature sensor. Figure 3 shows the structure design of the micro-heater and the temperature sensor. Table 2 shows the dimensions of the micro-heater and the temperature sensor of the PolyMUMPs sensors.

## 4. Layout and Fabrication of the PolyMUMPs Sensor

The layout of the PolyMUMPs gas sensors were carried out from the two-dimensional architecture of the designer tool of the CoventorWare software based on the PolyMUMPs process after obtaining the optimal design. CoventorWare is a software that has been used to design, simulate, and adapt MEMS devices by utilizing finite element analysis (FEA) and an appropriate-sized mesh element to test the device’s characteristics. CoventorWare is a virtual platform that simulates the real-world design environment in order to decrease fabrication cost and time, and any design flaws, by anticipating and rectifying any design faults using FEA. The PolyMUMPs technology’s final layout is exported to a GDS format before being transmitted to MEMSCAP US for fabrication with a die dimension of 10 × 10 mm.

**Figure 4:**
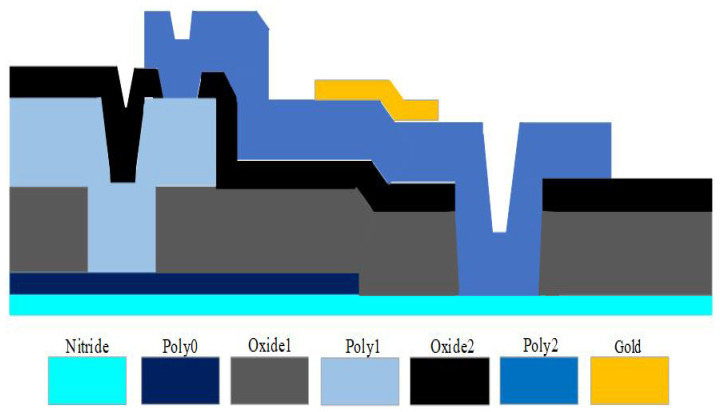
Cross-sectional sketch for the seven layers of the PolyMUMPs technology.

MEMSCAP provides cost-effective, proof-of-concept MEMS fabrication to universities, industries, and governments. The standard PolyMUMPs technology uses a surface micro-machining process and consists of different components that include single-crystal silicon utilized as a device substrate, silicon nitride used as a structural anchor, an electrical isolation between the polysilicon and the substrate, a polysilicon layer used as a structural material, and silicon dioxide used as a sacrificial layer. Figure 4 shows the cross-sectional view for the seven layers of the PolyMUMPs process. MEMS devices were successfully fabricated at MEMSCAP US based on the standard PolyMUMPs technology. Table 3 shows the nominal thicknesses of these layers as well as their material properties [25].

The device’s structural layers in the PolyMUMPs technology are made up of three low-pressure chemical vapour deposition (LPCVD)-produced polysilicon layers. The polysilicon layer has 10 MPa of residual stress, 158 GPa of Young’s modulus, a 0.22 of Poisson’s ratio, and 2300 kg/$m^{3}$ of density. The thicknesses of these layers are 0.5 μm, 2.0 μm, and 1.5 μm, respectively.

**Figure 5:**
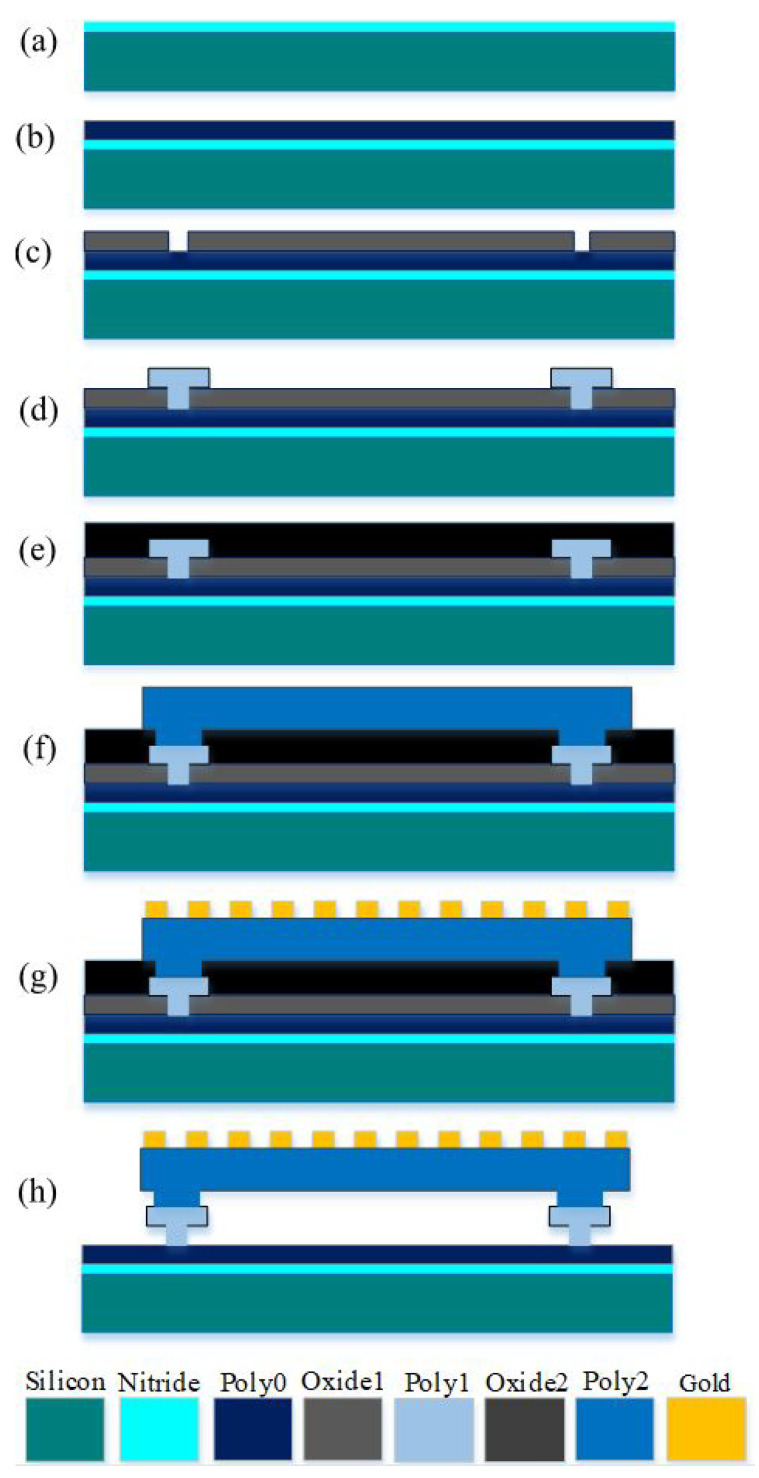
Cross-sectional view of the PolyMUMPs technology for the fabricated device shows (**a**) a silicon nitride deposition, (**b**) first polysilicon layer, (**c**) first sacrificial layer, (**d**) second polysilicon layer, (**e**) second sacrificial layer, (**f**) third polysilicon layer, (**g**) metal (Gold) layer deposited and (**h**) a final released structure.

Polysilicon 1 (Poly1) is not used in the device structure to provide a bigger gap between the stator polysilicon 0 (Poly0) and the moving plate polysilicon 2 (Poly2) for capacitance sensing. To produce the gap between the moving plate and the bottom plate, phosphor–silicate glass (PSG) oxide sacrificial layers with thicknesses of 2 μm (oxide 1) and 0.75 μm (oxide 2) are utilized (stator). The fabrication flowchart is illustrated in Figure 5 [25]. In this process, a 0.6 m silicon nitride was employed to separate the structure from the strongly doped substrate Figure 5a. To make the fixed plate, the first polysilicon layer is deposited and patterned (Figure 5b). Following this, the first sacrificial layer and the second polysilicon layer are deposited to construct the anchor parts, as illustrated in Figure 5c,d, respectively. The top plate is subsequently formed by depositing a second sacrificial layer and a third polysilicon layer, as shown in Figure 5e,f. On top of the Poly1 layer, a metal (Gold) layer is deposited and patterned to create a micro-heater and the temperature sensor. Because gold is conductive by nature, it is utilized for routing and as a pad material. MEMSCAP offers a dicing, releasing, and supercritical drying service to PolyMUMPs sensors. The structure is then released using 49% HF followed by carbon dioxide (${\mathrm{CO}}_{2}$) drying [25]. A schematic sketch of the final released structure of the device is shown in Figure 5g.

The summary of the various modeled dimensions compared to the measured dimensions of the fabricated devices are given in Table 4. The last column in the table indicates the percentage error.

**Figure 6:**
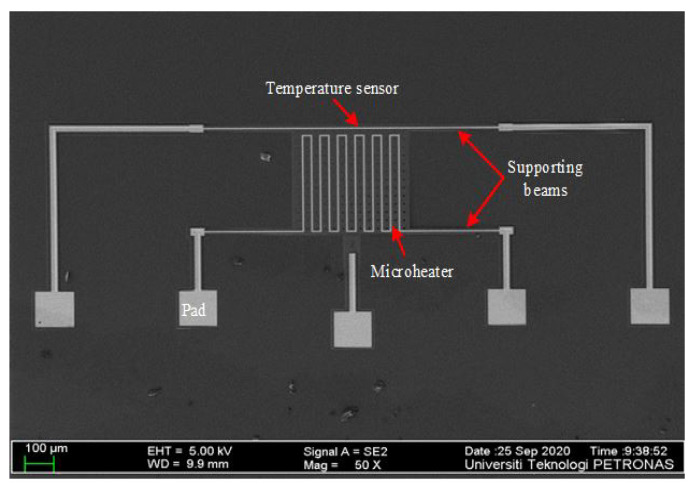
FESEM of the PolyMUMPs sensor showing measured plate parameters.

After fabrication, the received fabricated die was been imaged under an optical microscope to show the successfully fabricated device. Figure 6 shows the magnified view of the PolyMUMPs sensor imaged under field-emission scanning electron microscope (FESEM).

**Figure 7:**
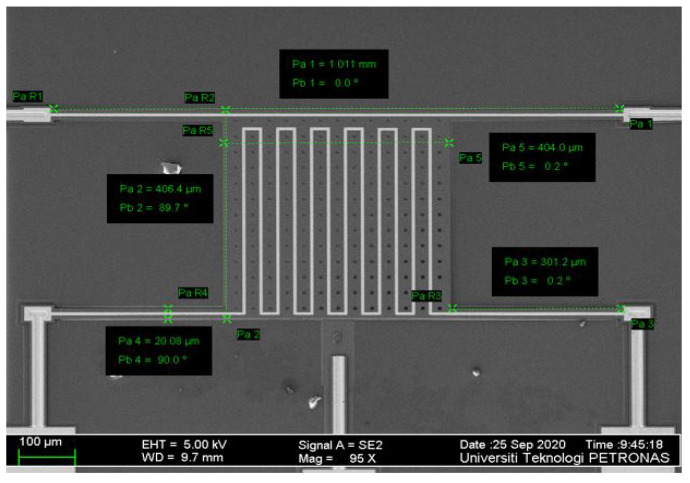
FESEM of the closeup view of the fabricated PolyMUMPs sensor.

The fabricated device matches the modeled and simulated one with variations in the thickness of the layers, which is probably due to the restricted angle of the measurement in the FESEM. Figure 7 shows the FESEM image with the measurement of the complete fabricated device. The total length of the fabricated device has a value of 1011 μm compared to the modeled length that was 1000 μm, showing a percentage difference of 1.09%.

The modeled length and width of the center plate are the same value of 400 μm as mentioned in Table 1, while the average length of the fabricated device is 404 μm and 406.4 μm, with percentage errors of 0.99% and 1.57%, respectively. The modeled length and width of each beam is 300 μm, while the average length of the fabricated device is 301.2 μm, with a percentage error of 0.40%. It can be noted that variation in the beam’s length will lead to a variation in resonance frequency. Increasing the center plate and the beam’s length will lead to increasing its mass and decreasing the frequency, and vice versa. It is noted that there are some differences between the measured and theoretical values, especially for the thickness of Poly2, Poly0, and gold, where the percentage error is high due to measurement angle inaccuracies from the FESEM machine.

**Figure 8:**
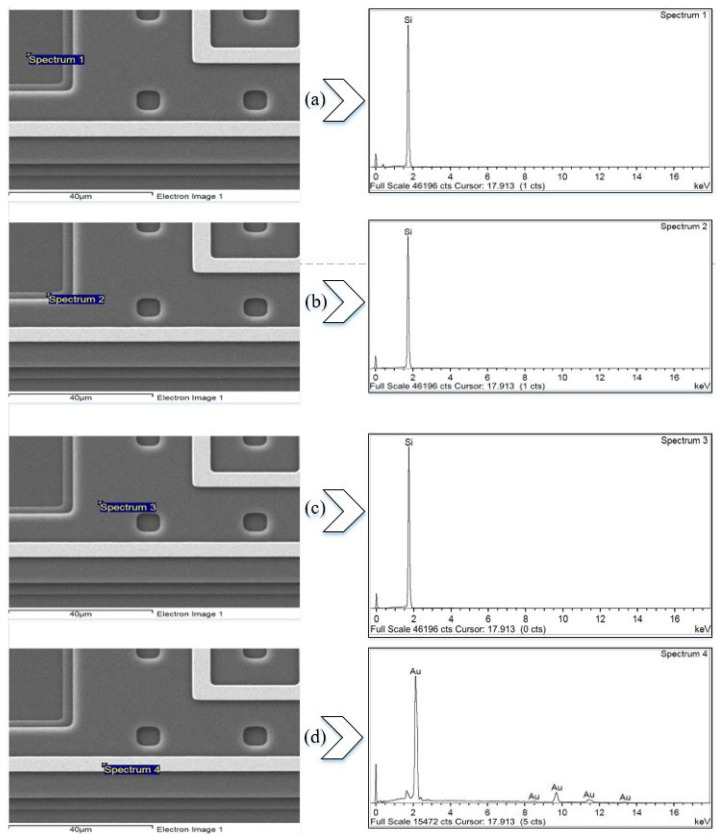
EDX analysis of the different layers of the PolyMUMPs sensor for (**a**) silicon substrate, (**b**) Poly0, (**c**) Poly2, and (**d**) gold.

Figure 8 shows the FESEM and energy-dispersive X-ray spectroscopy (EDX) for a section of the fabricated devices, confirming the materials of which the device is made. Figure 8a confirms the silicon nitride deposited on the substrate, while The bottom stator plate is Poly0, as in Figure 8b. The moving plate consists of Poly2, as confirmed by Figure 8c,d, which confirms that the micro-heater is made of gold.

## 5. Characterization of the PolyMUMPs Sensor

A probe station, power supply, humidity and temperature chamber, and some electrical equipment were used to characterize the PolyMUMPs sensor inside a clean booth with an area of 6 m × 4 m. The testing was conducted with an Agilent 34410A digital multi-meter and a GW Instek GPC-3030DQ power supply. Static characterization is used to evaluate the resistance values of the micro-heater and the temperature sensor compared to the modeled values prior to fabrication.

The experimental work starts with the connectivity measurement of the micro-heater and the temperature sensor resistance, followed by the assembling of the PolyMUMPs die to the printed circuit board (PCB). Then, the gold wire bonding to the PCB equipment was been conducted, while the last part investigates the temperature coefficient of the resistance measurement to determine the TCR under controlled temperature and humidity effects

### 5.1. Connectivity Measurement before Wire Bonding

**Figure 9:**
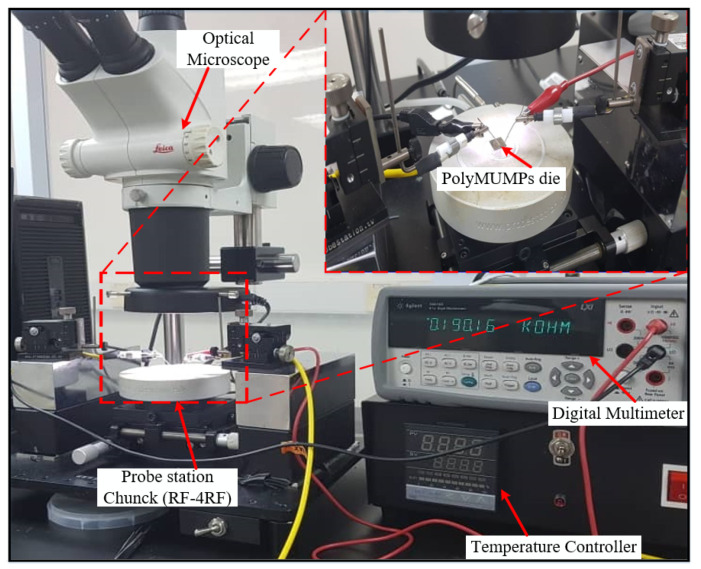
Electrical characterization of the PolyMUMPs sensor before wire bonding.

A connectivity measurement is important to measure the resistance values of the micro-heater and the temperature sensor of the fabricated device to ensure its connectivity. Before wire bonding, the whole die containing the devices is attached to the RF chunk using a photo-resist probe station (RE-4RF) to measure the connectivity of the device elements. The electrical connectivity for the micro-heater and the temperature sensor is determined through the measurement of their resistances, as shown in Figure 9. The RF probe station consists of a microscope, micro-positioners, chuck, an extension cable, a temperature controller, and four-probe needles. The PolyMUMPs sensor consists of five pads for interconnections with the outside world; two for the micro-heater for thermal actuation, two for the temperature sensor, and one connected to Poly0 to form the ground pad. To sense the capacitive changes, the parallel plates capacitor was performed by using the ground pad and one pad used from the temperature sensor on the top plate. The resistance of the micro-heater and the temperature sensor is measured using an Agilent (34410A) digital multi-meter (DMM) connected to the device pad terminals using two probe needles from the RF probe station. Figure 9 shows the characterization setup used to measure the resistance of the micro-heater and the temperature sensor.

The value of the measured resistances of the elements were compared to their theoretical calculated values, with the micro-heater having a measured resistance of 46.52 Ω at room temperature, showing a percentage difference of 0.727% with the theoretical value of 46.86 Ω, while the temperature sensor has a measured resistance of 9.04 Ω, showing a percentage difference of 0.893% compared to the theoretical value of 8.96 Ω.

### 5.2. The Printed Circuit Board (PCB) and WEST-BOND Gold Wire Bonder

After the connectivity test, the device was assembled on a printed circuit board. The fabricated devices on the PolyMUMPs die have their pads for electrical connection, but these pads are further needed to be connected to electrical equipment. The fabricated die is attached to the central part of the ceramic PCB using a Circuit Works conductive epoxy (silver epoxy). The conductive epoxy is made up of two components and can be used for bonding. It has excellent electrical conductivity and high strength. A hot plate is used to heat the PCB with the attached PolyMUMPs die to 90 °C for around 15 min to ensure an appropriate adherence.

The electrical connection from the fabricated device pads to the electrical equipment is achieved through wire-bonding equipment, which is a special type of equipment that can bond metal wires for electrical connectivity. Wire-bonding equipment is needed to make an electrical connection between the PCB chip and the MEMS devices’ external leads, which can be done with a wedge, ball, or ultrasonic bonding. These three types of bonding can be achieved with low-speed or manual wire bonders (typically in a research lab) and high-speed computer-controlled wire bonders (at industrial facilities). Bond wires usually consist of one of the following materials: gold (Au), copper (Cu), or aluminum (Al).

**Figure 10:**
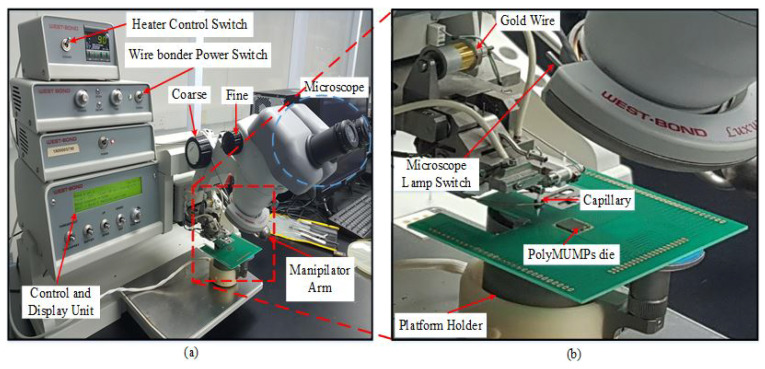
View of (**a**) WEST BOND gold wire-bonder model 7700E (**b**) side view of the gold-wire-bonding process.

WEST BOND gold-wire bonder (model 7700E) was used to bond the device. It consists of a microscope, platform holder, wire-bonder power switch, hot plate power switch, capillary, manipulator arm to control the bonding, and control unit, as shown in Figure 10. In our research lab, ball bonding is used to make the electrical interconnections between a die chip and the PCB, and gold wires are used as the leads. Ball bonding is mainly limited to gold and copper wire, and it usually demands the use of heat. The parameters of the WEST BOND 7700E wire-bonding machine are depicted in Table 5.

**Figure 11:**
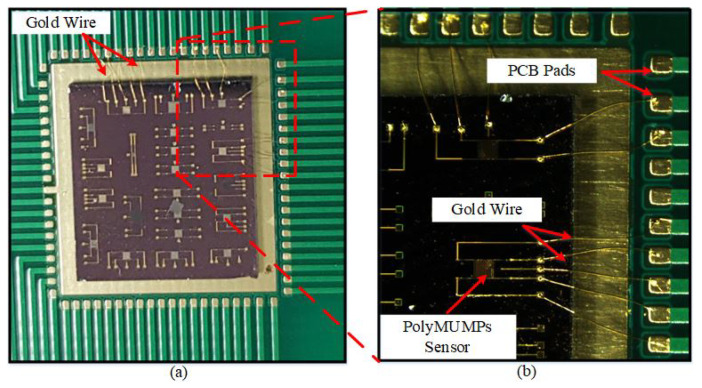
(**a**) Gold wires connecting sensors to the PCB pads, (**b**) a magnified view of a PolyMUMPs sensor wire bonded to PCB pads.

The PCB is placed on the heating-element holder of the wire bonder, with a pre-set holder temperature of approximately 96 °C. The various pads of the different components of the devices have been bonded onto the pads of the PCB. For wire bonding, it is important to heat the base, which makes the whole PCB and the die hot for the gold wire to connect properly. Figure 11a shows the gold wires connecting sensor pads to the PCB pads, and Figure 11b shows a magnified view of the two PolyMUMPs sensors that have bonded to PCB pads using gold wire.

### 5.3. Thermal Characterization of the PolyMUMPs Sensor

#### 5.3.1. Temperature Coefficient of Resistance (TCR) Measurement

**Figure 12:**
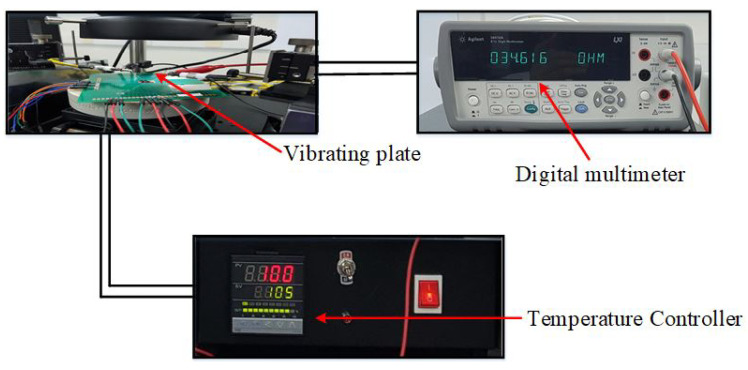
Block diagram for measuring temperature sensor resistance vs. ambient temperature.

The PolyMUMPs sensors were designed to use electro-thermal actuation and capacitive sensing. Hence, the thermal characterization was conducted to study the change of the micro-heater and the temperature sensor resistance due to the change of the environmental temperature. The temperature coefficient of resistance (TCR) of the sensor determines the rate at which temperature sensor resistance varies as a function of the environmental temperature; thus, the sensitivity of these sensors is dependent on this value [26]. To find the TCR of the temperature sensor and its relation to the environmental temperature, the block diagram of the system presented in the Figure 12 is used.

**Figure 13:**
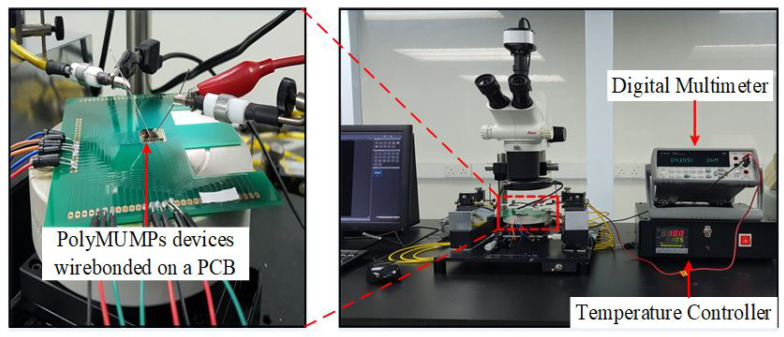
The setup for temperature versus resistance measurement.

To find the TCR of the temperature sensor, the temperature in a range of 25–110 °C has been applied on the vibrating plate, by increasing or decreasing the temperature of the probe station chuck in ±5 °C steps, as shown in Figure 13 [27]. By knowing and measuring the resistance increment or decrement due to the change in the temperature, the TCR can be calculated, and also the temperature can be calibrated using Equation (1) [28].

(1) $$\Delta R=\alpha_{T}R_{0}\Delta T$$

where ΔR denotes the change in temperature sensor resistance caused by a change in atmospheric temperature ΔT, and $\alpha_{T}$ denotes the temperature sensor material’s TCR (gold medal) [26].

The initial value of the measured temperature sensor resistance at 0 V is 17.822 Ω. The measured temperature sensor resistance is 18.216 Ω when applying 0.1 V, while it keeps rising to become 70.789 Ω at 1.6 V.

The resistance of the temperature sensor increases as the heating voltage increases, and the resistance has been determined with an Agilent digital multi-meter model 34410A. The voltage was adjusted in 0.1 V increments from 0.1 V to 1.6 V to achieve the same resistance change as direct heating with the probe station’s hot plate (chuck).

**Figure 14:**
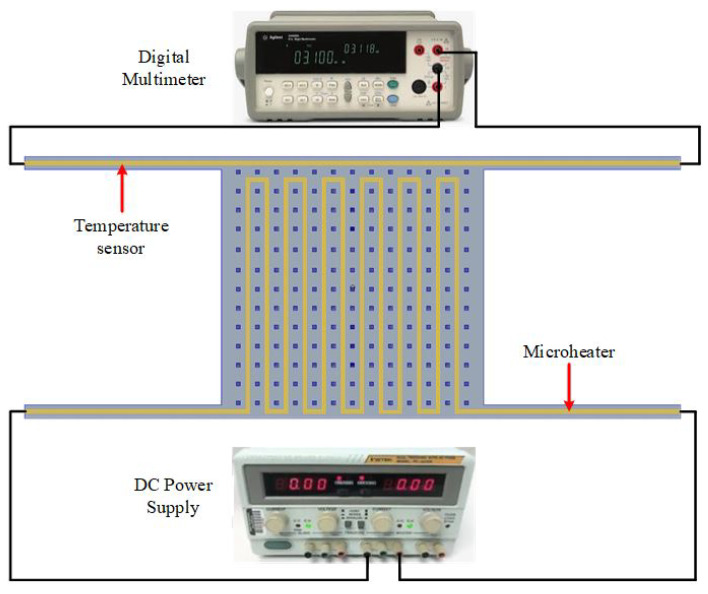
Schematic of the resistance versus voltage measurement.

The temperature is then calibrated using Equation (1). Using the diagram shown in Figure 14, the voltage was increased from 0.1 V to 1.6 V to achieve the resistance change required to obtain the temperature versus the applied voltage calibration curve.

**Figure 15:**
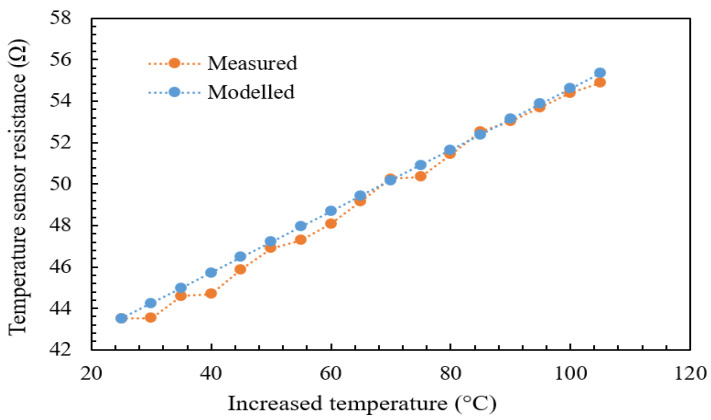
Modeled and experimental for the temperature sensor (gold) versus increasing temperature.

**Figure 16:**
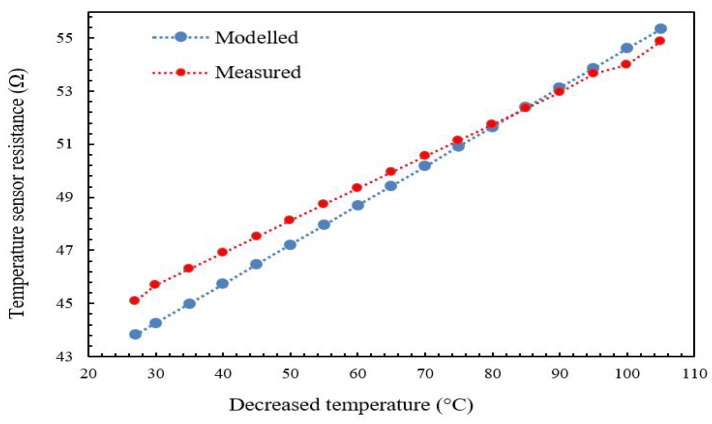
Modeled and experimental for the temperature sensor (gold) versus decreasing temperature.

Figure 15 and Figure 16 show the plot of the modeled and experimental resistance changes of the temperature sensor as a function of the increased plate temperature. The aim of measuring the value of the resistance change as a function of temperature change is to determine the temperature sensor’s TCR. The TCR can be obtained directly from Equation (1) if the resistance change is known. It is obvious that the relationship between temperature sensor resistance and plate temperature is linear with minimal hysteresis for increasing temperature regimes due to the positive TCR property of the gold. From the graphs produced using Equation (1), the TCR was observed to be in the range of 0.00325–0.0035 °C${}^{-1}$ when the temperature was raised from 25 °C to 110 °C, with a percentage difference of around 4.6%. The TCR was observed to be 0.00325–0.0035 °C${}^{-1}$ when the temperature was reduced from 110 °C to 25 °C, with a percentage difference of around 2.9%. The TCR standard value of gold (the material of the micro-heater and the temperature sensor) was reported to be 0.0034 °C${}^{-1}$ [14].

**Figure 17:**
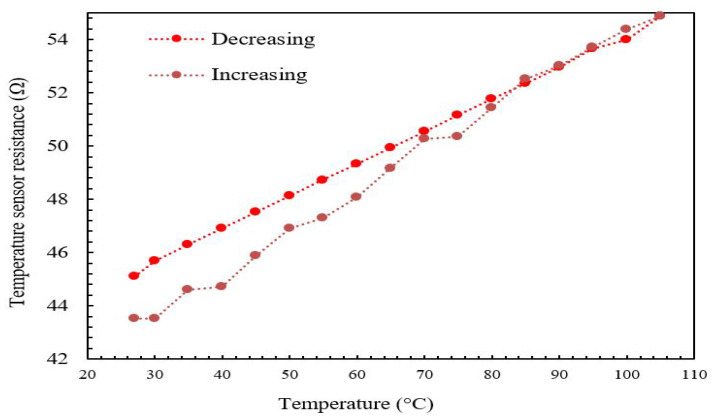
Experimental for the gold temperature sensor versus increasing and decreasing temperature.

The temperature sensor resistance varies linearly with minimum hysteresis as the temperature is varied (rising or falling) from 25 °C to 110 °C, as shown in Figure 17. The measured value of the TCR for gold is 0.00325 °C${}^{-1}$ and 0.0035 °C${}^{-1}$ for rising and falling temperatures, respectively. This agrees well with the reported value of α for gold, which is 0.0034 °C${}^{-1}$, with a change of 4.6% and 2.9%, respectively.

#### 5.3.2. TCR Measurement under Controlled Humidity

**Figure 18:**
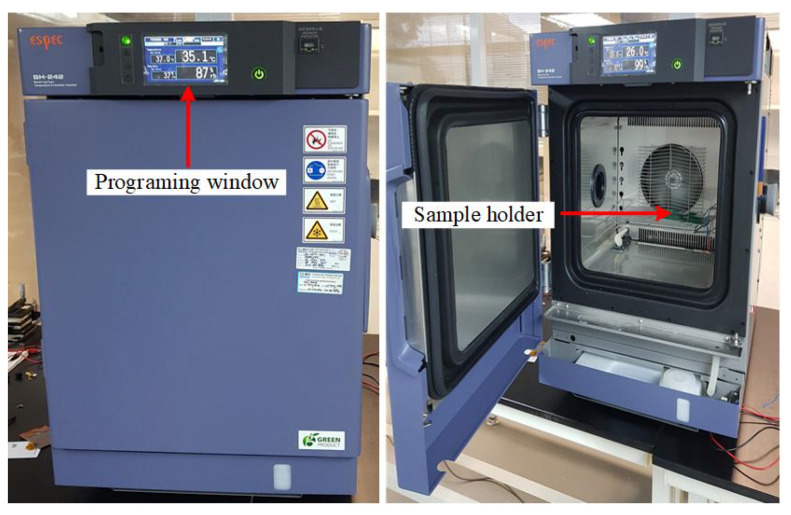
Closed and open SH-242 temperature and humidity chamber.

The temperature sensor of the fabricated PolyMUMPs sensor is characterized after wire bonding. The PolyMUMPs sensor was placed on a sample holder inside the SH-242 temperature and humidity chamber for TCR measurement. The specifications of the bench-top type SH-242 temperature and humidity chamber are listed in Table 6, while Figure 18 shows the photo of the closed and open temperature and humidity chamber of the bench-top type SH-242.

The temperature inside the SH-242 chamber was programmed to increase from room temperature (25 °C) to 110 °C in steps of 5 °C, and the resistance of the temperature sensor was recorded using an Agilent (34410A) digital multi-meter (DMM). Once the highest temperature of 110 °C was attained, the temperature of the sensor was reduced in 5 °C increments to room temperature (25 °C), repeating the same measurement.

**Figure 19:**
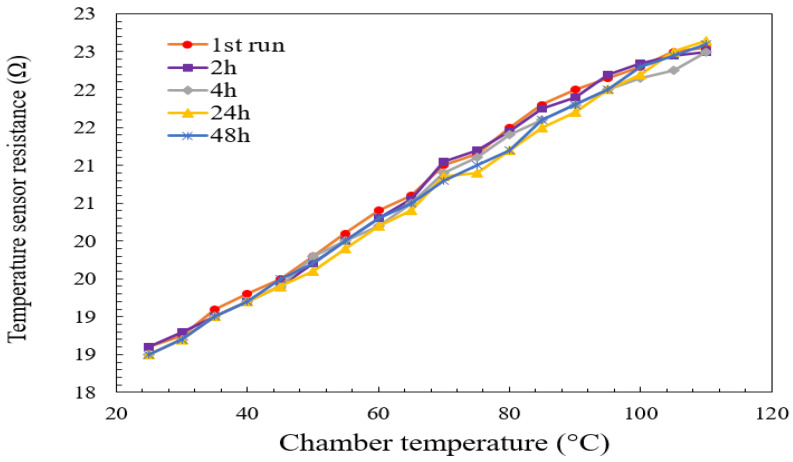
Constant measurement on the temperature sensor resistance versus chamber temperature at 60% RH.

For resistance repeatability, the test chamber was maintained at a constant relative the humidity of 60%, as the chamber temperature varied (rising or falling) from 25 °C to 110 °C. The value of the humidity was chosen to be 60% RH because the measurement of the humidity in the laboratory where the experiments were conducted was showing the same value. The test was conducted five times. The variation of the slope of the curves in Figure 19 was found to be negligible in all the tests. This figure contains five curves that overlap each other. Taking the first run as a reference, test two was performed after 2 h from the reference, while test three was performed after 4 h, and tests four and five were performed after 24 h and 48 h, respectively. The $R^{2}$ coefficient reached closer to 0.99, as can be seen in the Figure 19.

#### 5.3.3. Temperature Sensor Resistance vs. Micro-Heater Voltage Measurement

Heat was generated by supplying a DC voltage to the micro-heater of the PolyMUMPs sensor to investigate temperature sensor resistance against micro-heater voltage. A power source was used to apply a DC voltage to the PCB pins.

**Figure 20:**
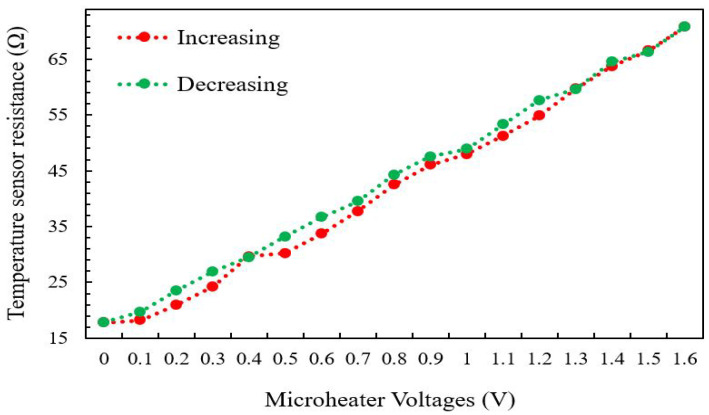
Temperature sensor resistance vs. increasing and decreasing of the micro-heater voltage.

The temperature of the micro-heater, as a result of Joule heating, was determined by measuring the temperature sensor resistance at various micro-heater heating voltages. The resistance of the temperature sensor has been measured using a digital multi-meter. Figure 20 shows how the temperature sensor resistance changes linearly as the micro-heater voltage is changed (increasing or lowering) from 0.1 V to 1.6 V, with a 0.1 V step.

#### 5.3.4. Gas Sensing Principle of the PolyMUMPs Sensor

**Figure 21:**
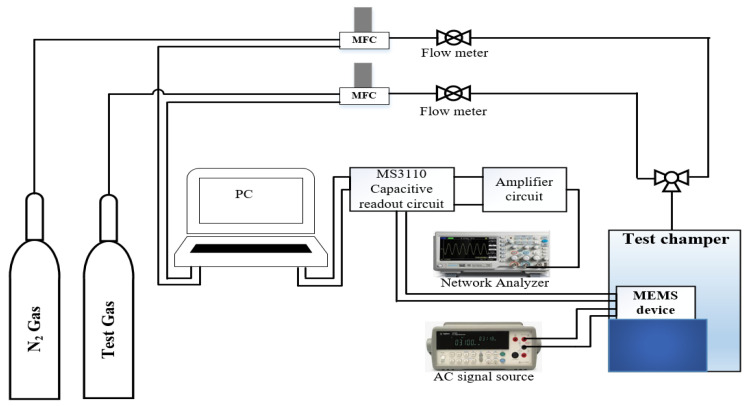
Block diagram of gas flow system.

To determine the sensor’s parameters, performance was evaluated for various concentrations of the targeted gas. The PolyMUMPs sensor was actuated at a certain frequency, and when exposed to an environment containing the desired gas, the detecting layer absorbed/adsorbed the gas. As a result of this interaction, the mass of the sensor increased, causing the displacement and/or frequency of the sensor to alter. This change is monitored and linked to the change in the targeted gas’ concentration. The gas flow system described in Figure 21 was used to produce the required concentration of the desired gas.

## 6. Conclusions

The temperature sensor and micro-heater of the PolyMUMPs sensor proposed for gas-sensing detection were fabricated and characterized in this paper. The micro-heater was designed to attain better temperature homogeneity with minimal power dissipation. The micro-heaters generate the required heat due to a certain current applied to the resistor that can result in low power consumption and a fast response time. The TCR of the sensor determines the rate whereby the temperature sensor resistance varies as a function of environmental temperature; thus, the sensitivity of these sensors is dependent on this value. The PolyMUMPs sensor is electro-thermally actuated and the output is capacitively detected. The micro-heater- and temperature sensor-measured resistance values were expected to be comparable to the modeled values with an acceptable error percentage of 5%. With a correlation value of 0.99, the temperature sensor resistance was observed to rise in a nonlinear relationship when the environmental temperature or the micro-heater voltage were increased. The TCR of the temperature sensor was determined to be 0.00325 °C${}^{-1}$ and 0.0035 °C${}^{-1}$, which included both rising and falling in a temperatures range of 25–110 °C, relative to the declared value of 0.0034 °C${}^{-1}$.

## Figures and Tables

**Table 1:** The PolyMUMPs sensor’s dimensions.

Parameter	Value (μm)
PolyMUMPs sensor length	1000
PolyMUMPs sensor width	400
Area of the center plate	400×400
The supporting beam length	300
The supporting beam width	20
The supporting beam thickness	1.5
The thickness of the movable plate	2
The vibration gap	4.75
The perforated holes dimension	6×6

**Table 2:** Dimensions of the micro-heater and the temperature sensor of PolyMUMPs sensors.

Parameter	Value	
	Micro-Heater	Temperature Sensor
Length (μm)	6880	1000
Width (μm)	5	5
Thickness (μm)	0.5	0.5
Resistivity (Ω.m)		$3.12\times 10^{-8}$
Thermal conductivity (Pw/μmk)		$2.97\times 10^{8}$
Thermal resistance (K/W)		$8.77\times 10^{4}$
Thermal mass (kg)		$4.17\times 10^{11}$
Heat capacity (J/K)		$5.39\times 10^{-9}$

**Table 3:** Material layer properties utilized in the PolyMUMPs process are different [25].

Layer	Thickness (μm)	Density (kg/m^3^)	Young Modulus (GPa)
Substrate (Si 100-N type)			
Silicon nitride	0.6	2300	130
Poly0	0.5	2700	254
Oxide 2	0.75	2230	158
Poly1	1.5	2230	158
Metal (Gold)	0.5	19,300	57

**Table 4:** Comparison of the theoretically modeled with the fabricated device parameters.

Parameter	Modelled Device (μm)	Fabricated Device (μm)	Percentage Errors (%)
Device length	1000	1011	1.09
Plate length	400	404	0.99
Plate width	400	406.4	1.57
Supporting beam length	300	301.2	0.40
Supporting beam width	20	20.08	0.40
Micro-heater wire spacing	24	23.69	1.29
Micro-heater length	5230	5255.2	0.48
Micro-heater width	5	5.51	9.26
Poly0 and Poly2 gap	4.75	4.43	6.74
Gold layer thickness	0.5	0.574	12.89
Poly2 layer thickness	1.5	1.256	19.43
Poly0 layer thickness	0.5	0.583	14.24
Etched hole length	6	6.33	5.21

**Table 5:** The parameters of the WEST BOND 7700E wire-bonding machine.

Parameters	Value
Vertical movement range (mm)	14.29
Horizontal movement range (mm)	15.88
Ultrasonic power supply (W)	3.25
Ultrasonic time (ms)	30
Wire feed angle (°)	45
Contact forces (gram force)	15∼250
Bonding wire, gold, diameter	20 μm
Work-piece temperature (°C)	96∼115 °C
Loop height before bond (μm)	2540
Drop before clamp (μm)	660
Motor steps for wire pull	34
Motor steps for wire tail	28

**Table 6:** Specification of the temperature and humidity chamber bench-top type SH-242.

Item	Value
Inner dimension (W×H×D)(mm)	300×300×250mm
Outer dimension (W×H×D)(mm)	440×630×730mm
Temperature chamber range	−40 to 150 °C
Humidity chamber range	30% RH to 95% RH

## Abbreviations

The following abbreviations are used in this manuscript:

MEMS	Micro-Electro-Mechanical System
PolyMUMPS	Polysilicon Multi-Users MEMS Process
